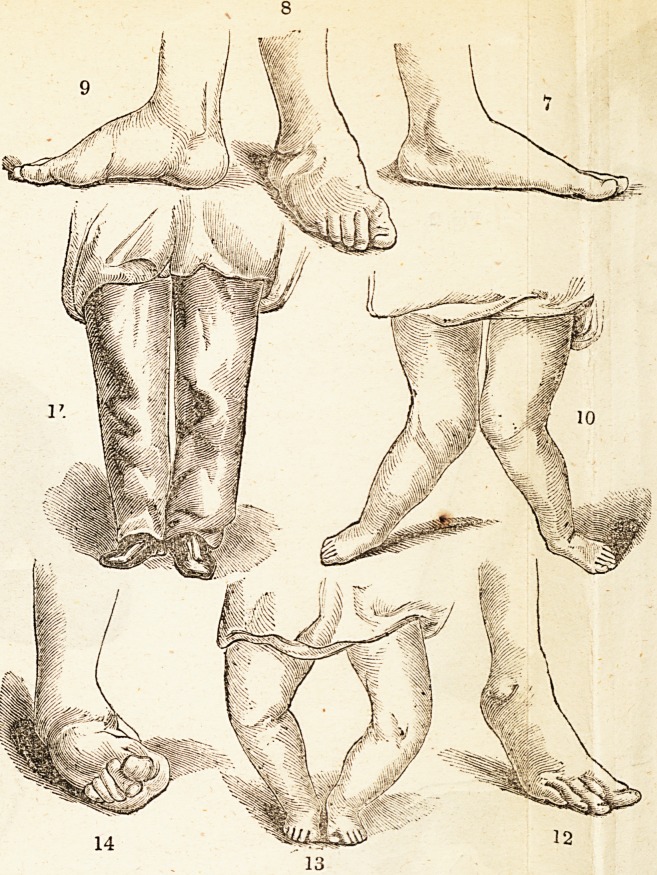# Patent Body Supports Invented by J. Amesbury, M.R.C.S. &c.

**Published:** 1840-04

**Authors:** 


Fig. 1.

DEFORMITIES *of the Spine, Chest, and Limbs;* MUSCULA[illegible]WEAKNESS; STIFF JOINTS and WEAK JOINTS—treated with the a[illegible]ance of "Patent Supports," and other contrivances, invented by J[illegible]EPH AMESBURY, Surgeon, M.R.C.S., late Lecturer on Orthope[illegible]c Surgery, &c.

---

Fig. 1 is a front view of the deformity existing in the son of a [illegible]ectable surgeon in Falmouth. The child was eight years old whe[illegible] was brought to Mr. Amesbury, and was very irritable, weak, and [illegible]ate. He had been using the ordinary means as directed by the hig[illegible]surgical authorities, but without any apparent beneficial effect. [illegible] de-

Fig. 2

forming process continued to increase rapidly. Mr. Amesbury had recourse to his Patent Spine Support, and Patent Exercising Plane, with manipulations and a generous diet. The curvature of the spine, which was similar to that seen in Fig. 3, yielded to these measures, and the body was brought up, and the figure restored to the extent seen in Fig. 2, in less than twelve months. The child was raised by the reduction of the curvature about three and a-half inches. His health has gradually improved, and he has become much stronger. He is still under treatment, for the removal of what remains of the curvature and deformity of the ribs.

Fig. 3.

Fig. 3 shows the deformity of the back in the daughter of a respectable tradesman, placed under Mr. Amesbury's care when she was eight years old. The deformity commmenced in infancy, and continued to progress rapidly, resisting and overcoming the means made use of for its removal. The deformity appeared, when viewed in front, of the same description as that seen in Fig. 1, but it had proceeded to a much greater extent. Her health was very ailing, and her temper irritable. Mr. Amesbury employed his "Patent Exercising Plane," and "Patent Spine Support," and manipulated the deformed parts from time to time. The curvature gave way under the operation of these means, so that in the course of about nine months the child was gradually raised by the reduction of the curvature upwards of five and a half inches. Her health

Fig. 4.

and strength improved with the improvement in her figure, and her mind became more easy and tranquil.

Fig 4 is a front view of the chest in this patient, as it appeared about fifteen months after the commencement of the treatment above mentioned. This child is still under Mr. Amesbury's care, in one of his private Orthopedic Establishments,* for the removal of the remaining defects in the figure of the chest and spine.

* These establishments are conducted by Mr. Amesbury, and are in separate houses, appropriately arranged and classed with due regard to *sex, rank, and age*, and are for the reception, accommodation, relief, and cure of persons afflicted with DEFORMITY of the *Spine, Chest and Limbs, General Muscular Weakness, Partial Muscular Weakness, Stiff Joints and Weak Joints.* Mr. Amesbury may be seen at his house, No. 59, Burton Crescent, Mondays, Wednesdays, Fridays, and Saturdays, from *ten* till *twelve* o'clock.

Fig. 5.

Fig 5. A case of deformity of the chest, occurring in a rickety child now under Mr. Amesbury's care. The sides of the chest were much flattened, and the lower parts preternaturally enlarged. The abdomen was greatly distended from the influence of weakness and disease. The state of the chest and abdomen, seen in this figure, is very common in children in slighter degrees. If proper curative means be not employed, the deformity generally becomes permanent, or goes on to the destruction of life. It readily yields, however, to the influence of Mr. Amesbury's "Patent Supports," with occasional medical treatment, manipulations, and a generous diet, as seen in the following case, Fig. 6.

Fig. 6.

Fig. 6 represents the improved state of Miss ———, aged two years, who was brought to Mr. Amesbury in a very weak condition, with the chest deformed and the abdomen enlarged, in the manner and to the extent seen in Fig. 5, but in her case there was also deformity of the spine. The curvature of the spine was reduced, and her figure restored in the manner represented in this plate, by manipulations and the use of Mr. Amesbury's "Patent Support," with occasional appropriate medicines, and a generous diet.

Fig. 7. An inside view of the left foot of a young gentleman, in which is seen that variety of deformity called *flat-foot*. Fig. 8. An outside and front view of the right foot, in which is seen the same deformity. This deformity is very common in various degrees, sometimes with and sometimes without inversion of the ankle-joint. The ankle-joint, however, usually projects unnaturally inwards over the inner side of the shoe, and the deformity is accompanied with more or less weakness of the feet and ankles, and a degree of awkwardness in walking, which is sometimes very great. Fig. 9 represents a foot in its natural arched form, showing the condition into which it is brought by the means which Mr. Amesbury employs.

Fig. 10 shows a case of *flat-foot, in-ankle,* and *in-knee,* existing in both limbs in the same child. This child now stands with the ankles and knees together, as seen in Fig. 11. The deformity commenced first in the feet, producing *flat-feet;* as the deforming process proceeded, the ankles were turned inward, and then the knees, so that when the child was brought to Mr. Amesbury, the feet were eight inches apart when the child stood with the knees together, as is seen in Fig. 10. The drawing, Fig 11, represents the child as he appeared dressed over Mr. Amesbury's Supports. In this case the limbs were brought from the position seen in Fig. 10 to the position seen in Fig. 11 in about a *fortnight* from the first application of the Supports.

Fig. 12 represents a case of *pointed toe.* This deformity commonly takes place after birth, and is usually the result of partial weakness, but sometimes it occurs as the consequence of disease or accident.

Fig. 13 shows the deformity commonly called *bow-knee,* or *out-knee.* This, like the deformity seen in Fig. 10 occurs sometimes in one knee, sometimes in both; occasionally one knee is bent inwards and the other outwards. This deformity takes place after birth, and is the result of partial, and sometimes of general, weakness in the body.

Fig. 14 shows a case of *out-ankle,* a variety of deformity which varies considerably in different persons, and is very frequently observed at birth; but it occasionally arises also from weakness of the ankle-joint

---

### REMARKS.

The several kinds of deformity herein shown, admit of relief, and frequently if treatment be commenced sufficiently early, of permanent cure without any distress or injury to the persons in whom they exist.

The cases here mentioned by Mr. A. had been suffered to go on to a very aggravated state, but notwithstanding the difficulties he had to encounter, the effects of the treatment adopted by him have been very satisfactory.

The great object which a surgeon should have in view in the treatment of the various deformities, &c., which occur in the human body is to restore the affected parts to their natural relative positions and functions, without distress to the patient, and without depriving the body of free exercise during the progress of restoration, as this is found to be of the greatest importance for the maintenance of health, and for the proper development of the human form. This principle has been borne in mind by Mr. A. in the construction and use of the various "*Supports*" and other means which he employs in the treatment of these cases. His Supports continue their beneficial operation in sustaining and protecting the deformed or weak parts under all the varying positions of the body, whether the person be placed in the standing or lying, or any other posture, or taking exercise in the open air, or otherwise. These advantages are derivable, not only from the judicious management of the Supports which Mr. A. employs in the treatment of deformities and weaknesses of the limbs, but also from that which he uses in the treatment of curvatures and weaknesses of the *Spine.* Hence it will be seen that the long confinement in the inclined or horizontal posture which has been had recourse to, and considered by many to be necessary in the treatment of lateral spinal curvature, may now be discontinued, and all its attendant evils consequently avoided. In the treatment of curvatures of the spine, Mr. A. has recourse to the use of his "Patent Exercising Plane," and "Patent Spine Support," either separately or in connection, as the condition of the parts and other circumstances might indicate; and with these he employs such medical and other remedial agents as in his judgment are calculated to facilitate the restoration of the spine, chest, and limbs, and increase the natural powers of the patient; and in the m[illegible]

nagement of deformities of the limbs, weakness or stiffness, he uses such of his other inventions as are suited to assist him in accomplishing his purpose of relief or cure.

Mr. A.'s "Supports" are not only used with a curative intention, but also in certain cases with a view simply to give support, and to relieve the bodily weakness and sufferings of the wearer. For further particulars and illustrations of Mr. Amesbury's treatment in stiffness, weakness, or deformity of the Spine, Chest or Limbs, see

His Pamphlet on Deformities, &c., entitled

NOTICE OF PATENTS,

*Granted to* JOSEPH AMESBURY, *Surgeon, Burton Crescent, London,*

Published by Longman and Co., Paternoster Row, London, and may be had of all Booksellers; or the 1st Vol. of his work on DEFORMITIES, which is now in the press, and shortly will be published.

By the same Author,

PRACTICAL REMARKS on the NATURE and TREATMENT of FRACTURES of the TRUNK and EXTREMITIES; being the substance of that portion of his Surgical Lectures which relates to this subject. Illustrated by plates, wood-cuts, and cases. In 2 vols. 8vo., price 1*l.* 5*s.*

---

### PATENT BODY SUPPORTS,

Invented by J. AMESBURY, M.R.C.S., &c.

*Used instead of the ordinary Stays and Corsets, to sustain the natural Figure, &c.*

These "Supports," for which Mr. Amesbury has lately obtained patents, guard the spine, in a great measure, against *Lateral Curvature,* and tend to enlarge the *capacity of the chest.* They also combine the principle, and supply the place of *Abdominal Belts* and *Back Boards.* They are made principally in *three varieties, in order to suit the age and the varying condition of the body at all periods of life.*

*First Variety, called* "PATENT SIMPLE SUPPORT." This is worn before and after the body has done growing, to maintain the natural figure, and is more comfortable in use than any of the ordinary stays. Its employment is particularly called for where there is slight unnatural fulness of the abdomen, partial or general, and where there is unnatural roundness or projection of the shoulders, or any tendency to Curvature of the Spine.

*Second Variety, called* "PATENT REDUCING SUPPORT." This is used instead of the "Patent Simple Support," where there is unnatural fulness of the abdomen in a greater degree. This support is also employed for *curative purposes,* in cases where there is enlargement of the abdomen produced by disease; and also where the abdominal enlargement is accompanied with deformity of the chest, weakness of the back, or malposition of the shoulders.

*Third Variety, called* "PATENT ADJUSTABLE SUPPORT." This variety is constructed with a "*Patent Adjustable Busk,*" and it allows of being easily adapted, from time to time, to suit the increasing or diminishing size of the abdomen of the wearer. It is especially made for the use of Ladies during pregnancy, and after delivery. In extreme cases of enlargement of the abdomen, either general or partial, it sustains the parts comfortably, and may, like the above-named varieties, which are used in slighter cases of a similar description, be also employed to produce a curative influence.

These "Patent Body Supports" are easily adjusted by the Wearers, except when had recourse to to remove some unnatural swelling, or to correct some defect; in such cases their application should be regulated by a competent medical practitioner.

It is to be observed that, in cases of confirmed curvature of the spine, the Inventor employs his "Patent Spine Support," and other appropriate means, and NOT his "Patent Body Supports;" and in cases of pregnancy, accompanied with curvature of the spine, he uses his "Patent Spine Support" in combination with parts of his "Patent Adjustable Support." The above Supports are stamped with the Royal Arms, and Mr. Amesbury's name and device. Pamphlets respecting their application and use may be had of his AGENTS in town and country.

THOMAS LAYTON, *Chief Manager.*

*** Ladies residing in London may be waited upon by application to Miss Wilkins, Factory, No. 8, Berners Street, Oxford Street.

## Figures and Tables

**Fig. 1. f1:**
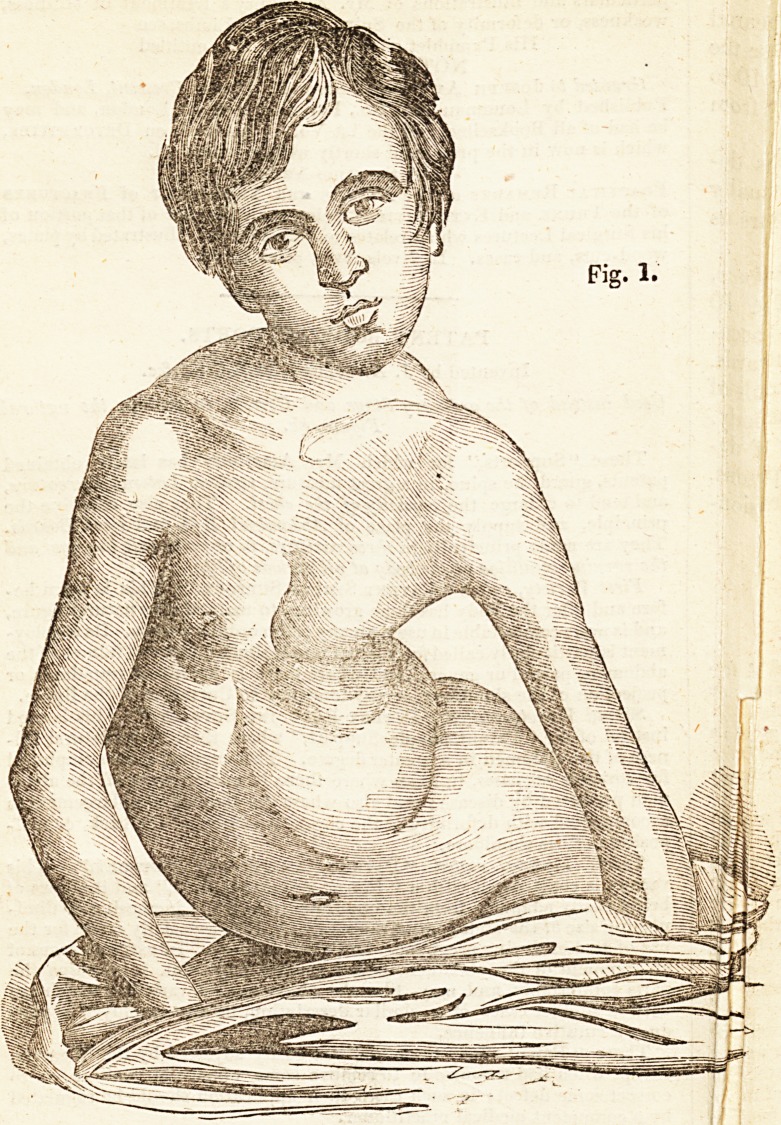


**Fig. 2. f2:**
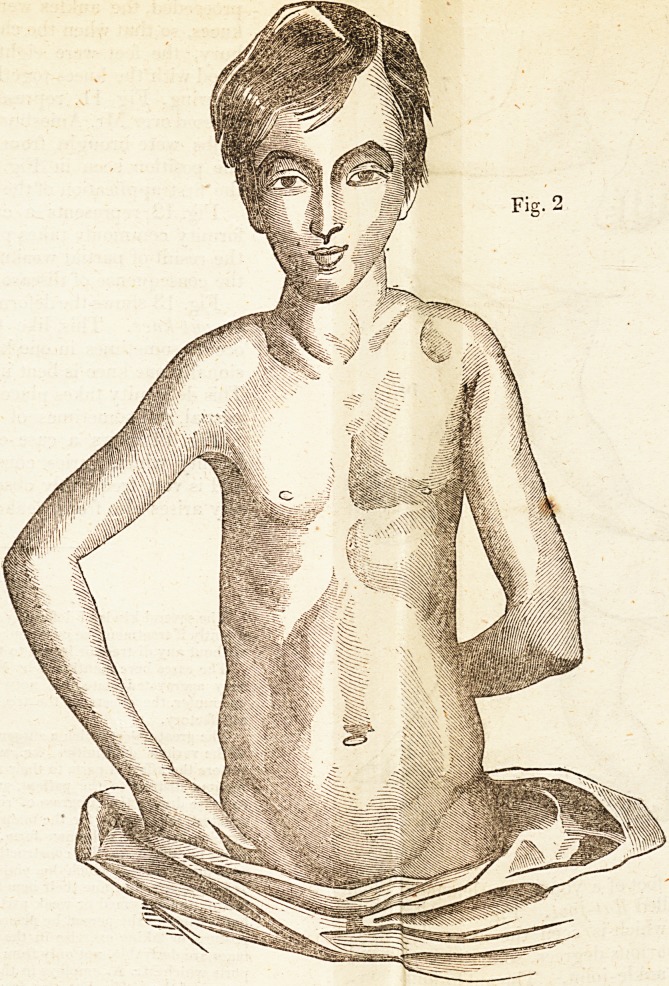


**Fig. 3. f3:**
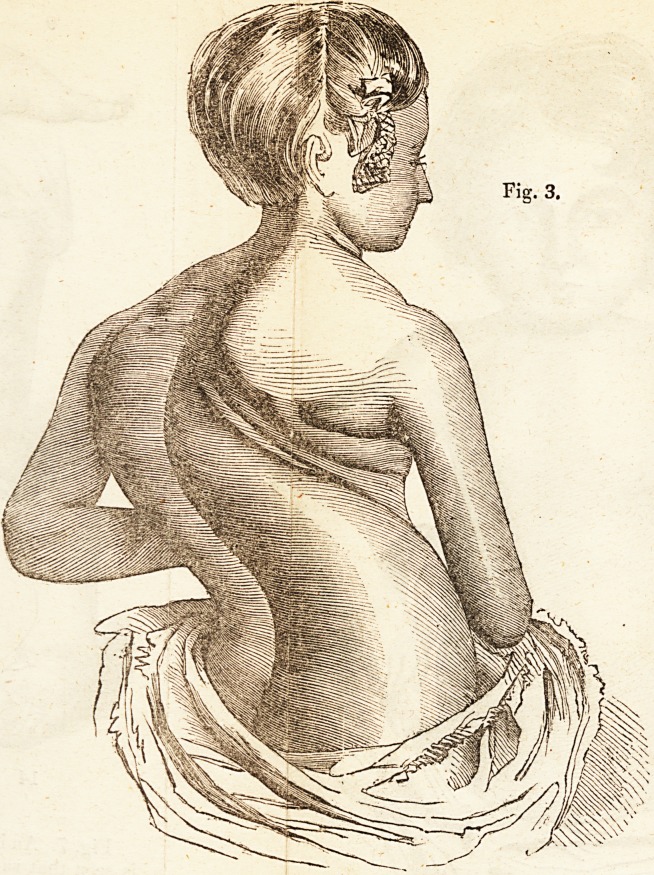


**Fig. 4. f4:**
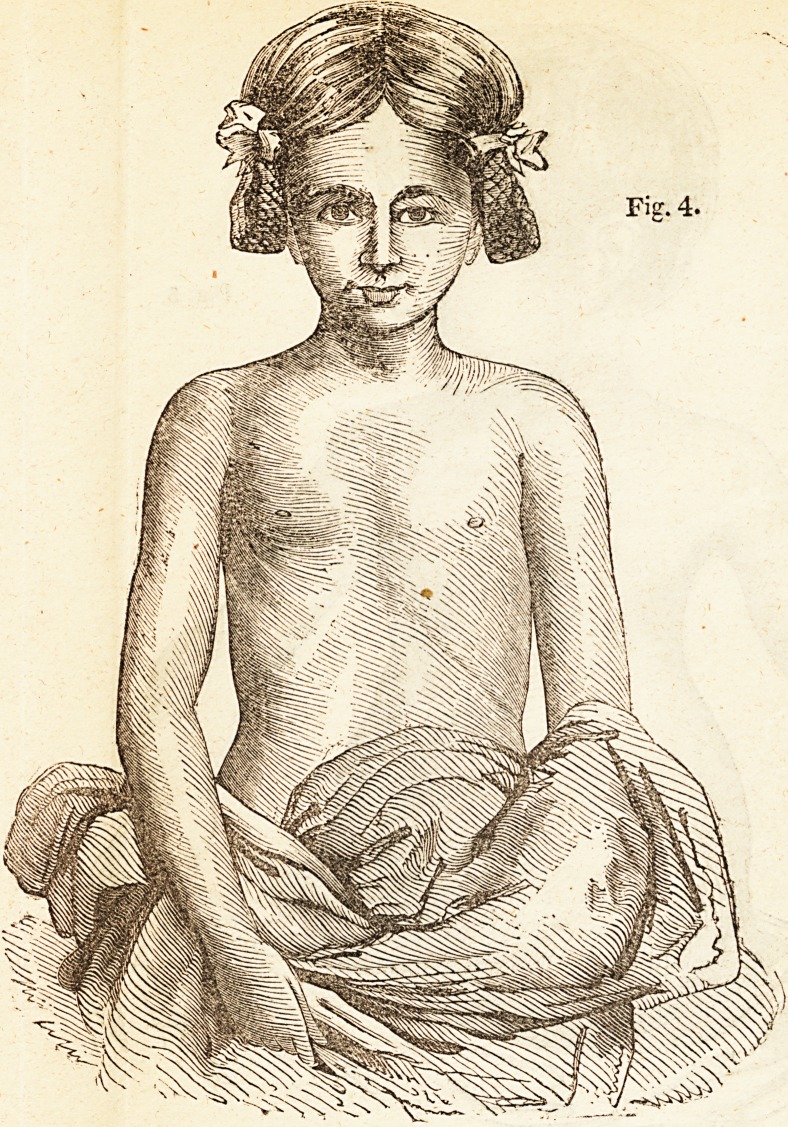


**Fig. 5. f5:**
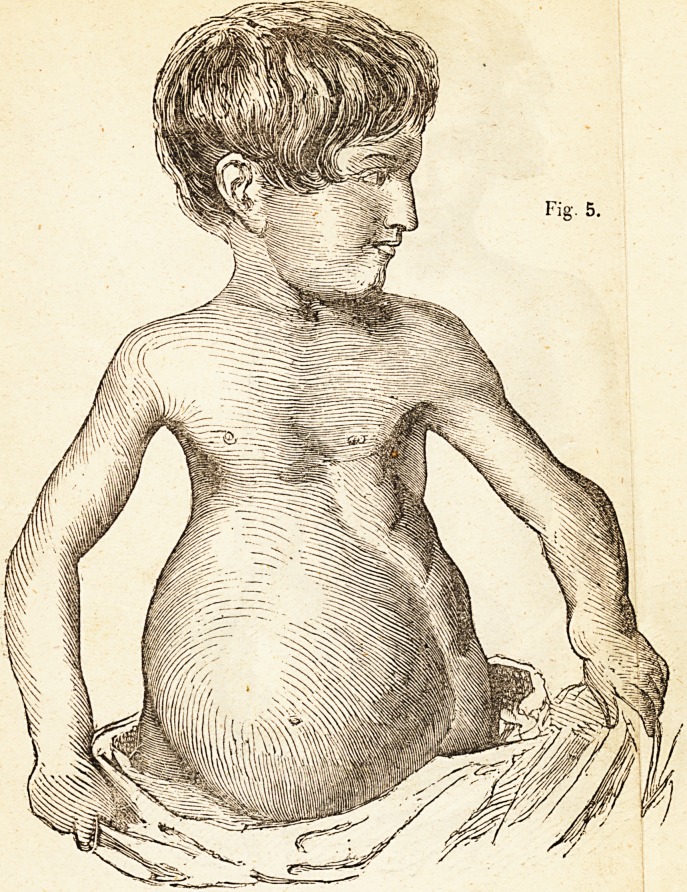


**Fig. 6. f6:**
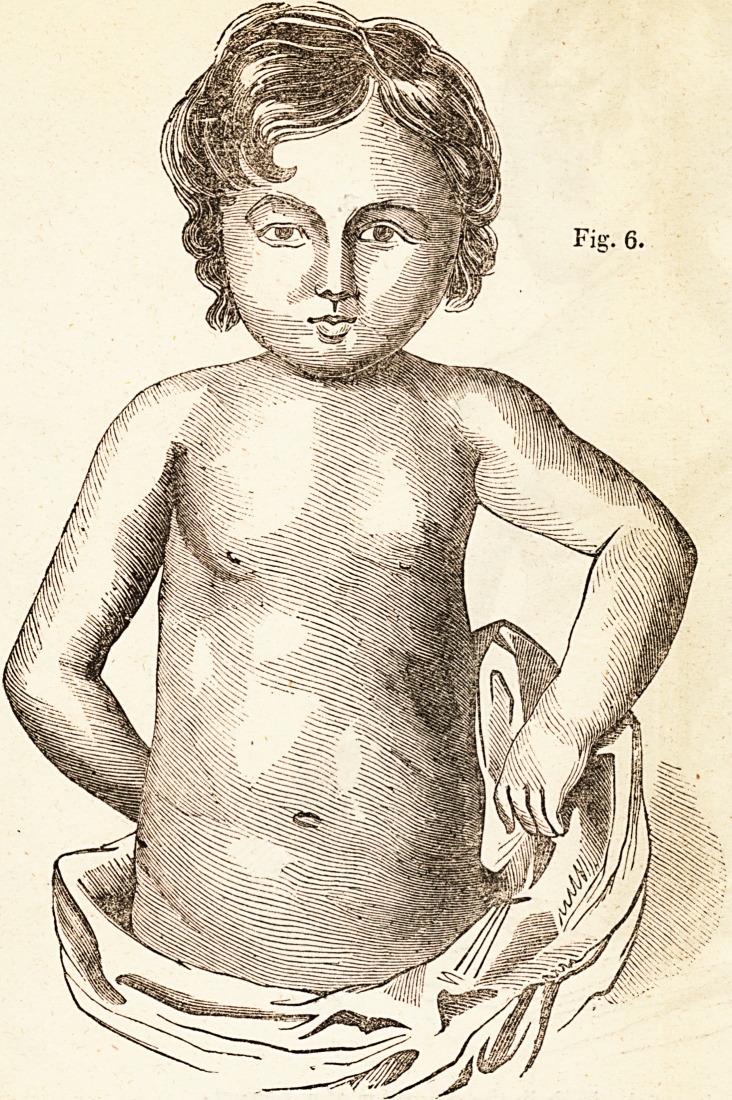


**Figure f7:**